# Energy Homeostasis and Abnormal RNA Metabolism in Amyotrophic Lateral Sclerosis

**DOI:** 10.3389/fncel.2017.00126

**Published:** 2017-05-04

**Authors:** Yu-Ju Liu, Po-Yi Tsai, Yijuang Chern

**Affiliations:** Division of Neuroscience, Institute of Biomedical Sciences, Academia SinicaTaipei, Taiwan

**Keywords:** amyotrophic lateral sclerosis, AMPK, TDP-43, importin, RNA binding proteins

## Abstract

Amyotrophic lateral sclerosis (ALS) is a fatal motor neuron disease that is clinically characterized by progressive muscle weakness and impaired voluntary movement due to the loss of motor neurons in the brain, brain stem and spinal cord. To date, no effective treatment is available. Ample evidence suggests that impaired RNA homeostasis and abnormal energy status are two major pathogenesis pathways in ALS. In the present review article, we focus on recent studies that report molecular insights of both pathways, and discuss the possibility that energy dysfunction might negatively regulate RNA homeostasis via the impairment of cytoplasmic-nuclear shuttling in motor neurons and subsequently contribute to the development of ALS.

## Introduction

Amyotrophic lateral sclerosis (ALS) is a rare motor neuron disease that occurs in adults with a prevalence of ~5 per 100,000. Patients with ALS develop muscle weakness and impaired voluntary movement, largely because of degeneration of the upper and lower motor neurons (Bento-Abreu et al., 2010; Kiernan et al., 2011; Al-Chalabi et al., 2016). Although substantial efforts have been made to investigate ALS pathogenesis in the past decade, no effective treatment is currently available. Most patients with ALS (~90%) have the sporadic form of the disease. Familial ALS (approximately 10% of cases) is caused by various mutations in more than 20 genes (Sreedharan and Brown, 2013; Steinberg et al., 2015; Taylor et al., 2016), including Cu-Zn superoxide dismutase (SOD1, Bruijn et al., 1998), TAR DNA-binding protein of 43 kDa (TDP-43, Neumann et al., 2006), fused in sarcoma/translocated in liposarcoma (FUS/TLS Lagier-Tourenne et al., 2010) and the expansion of (G4C2) repeat in C9ORF72 (DeJesus-Hernandez et al., 2011). The list is growing. It is intriguing that a significant portion of these ALS-associated genes are functionally important for the homeostasis of RNA- and DNA-binding proteins (Taylor et al., 2016). Another interesting observation is that abnormal energy metabolism (including weight loss, hyperlipidemia and mitochondrial dysfunction) has been observed in patients with ALS and in ALS mouse models, thus suggesting that abnormal energy homeostasis is an important hallmark of ALS (Ahmed et al., 2016). Moreover, abnormal activation of an energy sensor (AMP kinase, AMPK) is negatively associated with the survival of motor neurons and the impaired nuclear-cytoplasmic shuttling of ALS-associated genes/proteins (Lim et al., 2012; Liu et al., 2015a,b). This review is not intended to comprehensively describe RNA toxicity in ALS. Instead, it focuses on the emerging possibility that energy dysfunction in ALS might contribute to RNA toxicity and proposes a potential working hypothesis for the etiology of sporadic ALS.

## Abnormal Energy Homeostasis in AlS

### Body Weight, Hyperlipidemia and Energy Homeostasis in ALS

The balance between energy consumption and storage is critical in both healthy individuals, and individuals with diseases. There is substantial evidence demonstrating that energy homeostasis in patients with ALS is disrupted, because these patients usually exhibit a low body-mass index and abnormal energy metabolism (including hypermetabolism and hyperlipidemia), probably as a result of higher energy consumption and lower food intake (Desport et al., 1999; Vaisman et al., 2009; Dupuis et al., 2011). It is important to note that weight loss is negatively associated with survival and is therefore an important prognostic factor for patients with ALS (Stambler et al., 1998; Chio et al., 2009b; Jawaid et al., 2010). In addition, weight loss in patients with ALS is tightly linked to disease progression (Kasarskis et al., 1996; Desport et al., 1999, 2000). Weight loss in patients with ALS may be caused by dysphagia resulting from bulbar muscle weakness (Kühnlein et al., 2008). Another factor that may contribute to weight loss in patients with ALS is hypermetabolism. Given that patients with ALS have muscle weakness, and muscle tissue is a major site of energy consumption, hypermetabolism in patients with ALS is somewhat unexpected (Desport et al., 2001, 2005). Interestingly, patients with ALS have increased amounts of low-density lipoprotein (LDL), cholesterol and apolipoprotein E (APOE) in the blood (Lacomblez et al., 2002; Dupuis et al., 2008). Although hyperlipidemia in ALS is protective regarding respiratory function (Dorst et al., 2011), in a specific group of patients in Italy it has not been found to correlate with survival (Chio et al., 2009a). Lower body weight and increased metabolism, which occur before the onset of motor impairment, have also been observed in a widely used ALS mouse model that expresses disease-causing Cu/Zn-SOD1 mutations (i.e., G93A and G86R; Dupuis et al., 2004b). The causes of hypermetabolism and hyperlipidemia in ALS are currently unknown. It appears that the peripheral clearance of lipoproteins in ALS mice is enhanced (Fergani et al., 2007), suggesting that peripheral usage of lipids in ALS might be protective. Interestingly, feeding ALS mice (SOD1-G93A and SOD1-G86R) with a high-fat diet reduces the degeneration of motor neurons, delays disease onset and increases lifespan (Dupuis et al., 2004b; Mattson et al., 2007). Thus, therapeutic interventions for abnormal energy homeostasis in ALS may provide a strategy for the treatment of ALS.

Because some ALS patients tend to have higher levels of blood cholesterol and triglycerides (Dorst et al., 2011), the long-term effect of chronic administration of lipid-lowering drugs (such as statins) in patients with ALS has been a heated topic of debate in the past decade. Several studies reported a plausible association between chronic intake of statins and ALS (Edwards et al., 2007; Golomb et al., 2009). It was proposed that the detrimental effects of statins in patients with ALS might result from the suppression of cholesterol synthesis and the reduction of LDL availability to skeletal muscles. However, this claim regarding the association between ALS and statins has been contested (Colman et al., 2008; Sørensen and Lash, 2009) and requires further investigation.

To date, the mechanism underlying the association between abnormal metabolism and neurodegeneration remains to be determined. As Ahmed et al. (2016) have noted, it is important to clarify whether the atrophy of certain brain areas directly or indirectly affects metabolic homeostasis or if impaired metabolic metabolism causes degeneration of motor neurons.

## Aberrant Mitochondrial Function and AMP-Activated Protein Kinase (AMPK) Activation in ALS

Ample evidence has demonstrated that dysregulation of mitochondrial function is a central event in ALS pathogenesis and is closely associated with disease progression (Echaniz-Laguna et al., 2002; Crugnola et al., 2010). Mitochondrial dysfunction usually occurs via dysregulation of the electron transport chain machinery and/or decreased mitochondria cytochrome oxidase activity (Borthwick et al., 1999; Ricquier and Bouillaud, 2000), which significantly contributes to neuronal cell death (Gorman et al., 2000). Expression levels of some regulators of mitochondrial biogenesis (e.g., PGC-1α) are also decreased in the spinal cords and muscles of mice and patients with ALS (Thau et al., 2012).

### ALS Genes and Mitochondria

Many ALS-associated genes have been implicated in the regulation of mitochondrial function. For example, overexpression of mutant SOD1 (mSOD1) impairs mitochondrial function by disrupting the respiratory transport chain machinery (Beretta et al., 2003; Coussee et al., 2011), inhibiting the transportation of proteins into mitochondria by interacting with the mitochondrial channel protein (VDAC1, Israelson et al., 2010) and impairing mitochondria fission and fusion through alteration of the expression of DRP1 or OPA1 (Ferri et al., 2010; Liu et al., 2013). Furthermore, Wang et al. (2013) reported that overexpression of wildtype TDP43 or ALS-associated TDP-43 mutants causes the impairment of mitochondrial dynamics through increased localization of TDP-43 on mitochondria in motor neurons. FUS/TLS is also an important disease protein in ALS. Expression of FUS/TLS was found to trigger mitochondrial damage by interacting with the mitochondria chaperone protein, Hsp60 (Deng et al., 2015). Valosin-containing protein (VCP) is also involved in ALS pathology. Mutations in VCP cause mitochondrial dysfunctions, including impaired clearance of damaged mitochondria and decreased ATP levels (Chang et al., 2011; Kim et al., 2013). Recently, the C9ORF72 repeat expansion has been shown to increase oxygen consumption and mitochondrial hyperpolarization in patients with ALS (Onesto et al., 2016). iPSC-derived motor neurons from patients with a C9ORF72 repeat expansion also have a lower mitochondrial membrane potential than those from non-ALS controls, thus supporting the involvement of C9ORF72-induced mitochondrial dysfunction in ALS pathogenesis (Dafinca et al., 2016). Biochemical evidence suggests that the C9ORF72 repeat expansion causes mitochondrial damage by interacting with mitochondrial proteins, such as ATP synthase subunits alpha and beta (Rossi et al., 2015). Collectively, although via distinct pathways, mutations in multiple ALS genes result in mitochondrial dysfunctions that contribute to ALS pathogenesis. Improved mitochondrial functions as well as energy homeostasis in motor neurons and muscles have thus become the key therapeutic targets for ALS in recent years.

Beyond the degeneration of motor neurons, another major hallmark of ALS is muscle atrophy. Muscle atrophy is of great interest because the energy metabolism of skeletal muscle is an essential component of overall energy homeostasis. In an ALS mouse model, exogenous expression of human SOD1-G93A in the muscle induces severe muscle atrophy, reduced muscle strength and abnormal muscle structure (Dobrowolny et al., 2008). Similarly, overexpression of another disease-causing gene (TAR DNA-binding protein-43, TDP-43) also disturbs energy metabolism in mice by disrupting mitochondrial function and morphology in neurons (Shan et al., 2010; Xu et al., 2010). Genetic removal of TDP-43 also leads to defective energy homeostasis by decreasing adipose tissue (Chiang et al., 2010), suggesting that TDP-43 is involved in tightly tuned energy homeostasis, which is important in ALS. Collectively, these findings suggest that impairment of energy metabolism is a critical event in the pathogenesis of ALS. Further investigation is required to understand and block the pathogenic mechanisms underlying this energy dysfunction caused by mitochondrial defects.

### Mitochondria and AMP-Activated Protein Kinase (AMPK)

Outside of the mitochondria, the major component that regulates energy homeostasis at the cellular level is AMPK (Long and Zierath, 2006; Hardie, 2008), which has recently been implicated in ALS and other degenerative diseases. Specifically, abnormal AMPK activation was observed in Huntington’s disease (HD; Chou et al., 2005; Ju et al., 2011, 2012, 2014), Alzheimer’s disease (AD; Thornton et al., 2011; Mairet-Coello et al., 2013) and ALS (Lim et al., 2012; Liu et al., 2015a). The impact of AMPK activation during the progression of several neurodegenerative diseases (e.g., HD and AD) is seemingly complex and appears to depend on the disease stage and the specific context of the experimental model used in the study (Ju et al., 2011; Ma et al., 2014; Domise et al., 2016; Ng et al., 2016; Tulino et al., 2016; Vázquez-Manrique et al., 2016). In ALS, higher AMPK activity was found in the spinal cord of an ALS mouse model (SOD1-G93A; Lim et al., 2012). Suppression of AMPK activity either pharmacologically or genetically decreases the mSOD1-evoked death of motor neurons. Consistent with the hypothesis that abnormal activation of AMPK is detrimental, overexpression of a disease-causing SOD1 mutation (mSOD1) in *C. elegans* leads to locomotor dysfunction in an AMPK-dependent manner, whereas deletion of the ortholog of AMPK (i.e., aak-2) has been found to improve motor function in mSOD1-expressing or TDP-43 mutant-expressing worms (Lim et al., 2012). These findings were the first to suggest that abnormal AMPK activation, which is expected to cause energy dysregulation, may be relevant to ALS. Moreover, abnormal AMPK activation has been detected in motor neurons of patients with ALS and TDP-43-ALS mice and subsequently cause cytoplasmic mislocalization of TDP-43 and motor dysfunction (Liu et al., 2015a). Collectively, these results suggest that cellular energy status is critical for the function of motor neurons. Activity of the energy sensor AMPK appears to interfere with the proper cellular localization of an RNA-binding protein (TDP-43), which is considered an early event in ALS pathogenesis (Giordana et al., 2010). The loss of TDP-43 in nuclei due to its redistribution under stress and inclusion formation in the cytoplasmic region might, at least partly, contribute to the observed TDP-43 proteinopathy (Lagier-Tourenne et al., 2010).

Consistent with the abovementioned hypothesis that energy status is critical for ALS, previous studies have suggested that traumas affecting energy homeostasis can contribute to ALS pathogenesis. For example, hypoxia is a proposed risk factor for ALS (Vanacore et al., 2010). This possibility is interesting because hypoxia is known to induce AMPK activation via the production of reactive oxygen species (ROS) and downstream pathways (Mungai et al., 2011). Sufficient blood supply and oxygen availability to the spinal cord are critical. Lower neural vascular perfusion caused by the suppression of vascular endothelial growth factor (VEGF) induced degeneration of motor neurons in the spinal cord (Oosthuyse et al., 2001), while administration of VEGF increased the lifespan of an ALS mouse model (mSOD1, Azzouz et al., 2004). Beyond the importance of VEGF in the protection of motor neurons, these findings also support the hypothesis that proper maintenance of energy homeostasis is critical for ALS.

## Dysfunctional RNA Metabolism and Energy Homeostasis in ALS

Abnormal RNA metabolism may lead to RNA toxicity, which is usually caused by accumulation of toxic RNAs and dysfunction of RNA-binding proteins (Sicot and Gomes-Pereira, 2013). The latter has been well documented in various pathogenic events involving RNA metabolism, including RNA splicing, RNA localization, RNA transcription and miRNA biogenesis. Importantly, RNA toxicity has been implicated in various genetic diseases including fragile-X syndrome (Oberlé et al., 1991), spinobulbar muscular atrophy (La Spada et al., 1991), myotonic dystrophy type 1 (Brook et al., 1992) and HD (Bañez-Coronel et al., 2012). More recently, overexpansion of G4C2 hexanucleotide repeats in the noncoding region of C9ORF72 has been found in patients with ALS and frontotemporal lobar degeneration (FTLD; DeJesus-Hernandez et al., 2011; Renton et al., 2011; Gijselinck et al., 2012) and has been shown to play a critical role in ALS pathogenesis (Rohrer et al., 2015; Jiang et al., 2016; Lee et al., 2016; Lin et al., 2016; Taylor et al., 2016). In the following sections, we will focus on the literature suggesting potential aspects of RNA homeostasis that might be sensitive to impaired energy homeostasis in ALS.

### ALS Genes and Stress Granules (SGs)

Many ALS genes (e.g., TDP-43, FUS/TLS, hnRNPA1, MATR3) encode RNA-binding proteins that have been found to affect multiple levels of RNA processing, including mRNA transcription, RNA transport, mRNA stabilization and miRNA biogenesis (Ling et al., 2013; Taylor et al., 2016). Recent studies have suggested that disturbed RNA homeostasis is a central pathogenic pathway in ALS (Weishaupt et al., 2016).

Importantly, several ALS-related RNA-binding proteins are found in stress granules (SGs; Colombrita et al., 2009; Dewey et al., 2011; Bentmann et al., 2012; Li et al., 2013). Eukaryotic cells commonly respond to stress by forming SGs, which are composed of untranslating messenger ribonucleoproteins (mRNPs) and a variety of other proteins. The key function of SGs is to temporally inhibit translation and store mRNA during stress. It has been well documented that SGs recruit RNA-binding proteins and various RNAs, thus controlling the post-transcriptional regulation of RNA, RNA stability and translation. Translation initiation of mRNA-protein complexes existing in SGs is usually limited (Buchan and Parker, 2009; Jain et al., 2016). It has been proposed that the formation of SGs is closely associated with neurodegenerative diseases, because expression of disease-causing proteins (e.g., TDP-43, FUS/TLS) facilitates and/or sustains the formation of SGs (Andersson et al., 2008; Colombrita et al., 2009; Dewey et al., 2011; Bentmann et al., 2012; Li et al., 2013; Ramaswami et al., 2013; Aulas and Vande Velde, 2015). The dynamic state of SGs can be regulated by various stressors via energy-dependent pathways involving ATP-dependent remodeling complexes (Protter and Parker, 2016).

An earlier study has suggested that TDP-43 modulates the formation of SGs by controlling the amount of SG proteins (i.e., G3BP1 and TIA-1; McDonald et al., 2011). Exogenous overexpression of ALS-associated TDP-43 mutants enhanced the number and size of SGs when compared with wild-type TDP-43 (Liu-Yesucevitz et al., 2010). Similarly, FUS/TLS is also localized or recruited to SGs during stress exposure (Sama et al., 2013; Lenzi et al., 2015). A few ALS-associated FUS/TLS mutants were shown to affect the assembly and dynamics of SGs and might contribute to the pathogenesis of ALS (Baron et al., 2013). The detailed roles of TDP-43 and FUS/TLS in regulating SGs appear different and will be discussed in more detail in the following sections.

An interesting question emerging from recent studies is whether the TDP-43 inclusions observed in ALS motor neurons are derived from SGs (Li et al., 2013; Aulas and Vande Velde, 2015). This is an important question because multiple laboratories have reported that, after exposure to various stresses (including ER stress, mitochondrial stress and proteasome inhibition), TDP-43 is recruited to SGs (Colombrita et al., 2009; Volkening et al., 2009; Dewey et al., 2011). Once accumulated in SGs, TDP-43 and FUS/TLS may gradually evolve into stable protein aggregates as observed in diseased motor neurons (Parker et al., 2012). Moreover, mislocalization of FUS/TLS and TDP-43 in the cytoplasm is likely to recruit and sequester their interacting proteins into either SGs or inclusions and subsequently lead to disease pathogenesis induced by loss-of-function in motor neurons (Kamelgarn et al., 2016). Mislocalization of RNA-binding proteins (such as TDP-43 and FUS/TLS) and the formation/assembly of SGs have therefore attracted a great deal of attention in ALS research in recent years. Of note, because not all stress models show SG formation (Takahashi et al., 2013) and SG formation may be protective for cells (Anderson and Kedersha, 2008), whether the formation of TDP-43 inclusion is detrimental remains unknown (Baloh, 2011; Cragnaz et al., 2014), and the detailed function and regulation of SGs in ALS requires continued investigation.

In the following section, we will focus on the roles of TDP-43, FUS/TLS and the C9ORF72 hexanucleotide repeat expansion in the regulation of RNA metabolism in ALS. Although the compositions and properties of SGs have not been well characterized, the discovery of AMPK holoenzymes in SGs is intriguing (Mahboubi et al., 2015a,b). The abnormalities of energy metabolism and its downstream signals in modulating RNA homeostasis will also be discussed.

### TDP-43

In ALS, TDP-43 is a key pathological protein that forms cytosolic aggregates. TDP-43 consists of 414 amino acids and contains two RNA recognition motifs, RRM1 and RRM2. It is normally localized to nuclei and affects several RNA-related processes, including RNA splicing, RNA transcription, RNA transport and miRNA production. Previous findings have indicated that TDP-43 binds to more than 6000 RNA targets in the brain (Tollervey et al., 2011; Colombrita et al., 2012; Polymenidou et al., 2012). Earlier studies have suggested that TDP-43 is a transcriptional regulator that regulates the expression of many proteins (including CHMP2B, OPTN, VAPB and VCP) that are involved in protein homeostasis (Polymenidou et al., 2011) and in autophagy (e.g., the autophagy-related 7, Bose et al., 2011). TDP-43 also affects RNA stability by binding to the 3′-untranslated regions (3′UTRs) of specific mRNAs and regulating mRNA degradation (Polymenidou et al., 2011). Neuronal activation induces the translocation of TDP-43 to dendrites, where TDP-43 regulates RNA targets critical for synaptic functions (Wang et al., 2008).

In addition, TDP-43 is involved in miRNA biogenesis (Kawahara and Mieda-Sato, 2012). For example, down-regulation of TDP-43 increases the expression of miR-633 but decreases that of let-7b miRNA (Buratti et al., 2010). TDP-43 also controls the splicing pattern of many important genes, including amyloid beta precursor protein (APP), presenilin, huntingtin, multiple ataxins, α-synuclein, progranulin, FUS/TLS and TDP-43 itself (Polymenidou et al., 2011; Sephton et al., 2011). In addition, TDP-43 binds to long (>200 base) non-coding RNAs (ncRNAs), including the two highly expressed ncRNAs, nuclear-enriched autosomal transcript 1 (NEAT1) and metastasis-associated lung adenocarcinoma transcript 1 (MALAT1; Tollervey et al., 2011). Although the functional roles of this interaction remain unclear, both of these ncRNAs appear to be important because their levels are increased in patients with FTLD and ALS (Tollervey et al., 2011; Nishimoto et al., 2013). In post-mortem cortical tissues of FTLD patients, the amount of NEAT1 that binds to TDP-43 is also enhanced (Tollervey et al., 2011). NEAT1 is a component of a subnuclear structure (i.e., paraspeckles, huge RNP particles) and functions as a scaffold for RNA-binding proteins and RNAs. The functions of paraspeckles have not been clearly defined yet. Previous studies suggest that paraspeckles may be involved in the nucleocytoplasmic shuttling of specific mRNAs, transcription and pre-miRNA splicing under pathophysiological conditions (Naganuma and Hirose, 2013). Analyses of the spinal cords of ALS patients have shown that paraspeckles with high levels of NEAT1 and TDP-43 are found primarily in motor neurons of patients with early-stage ALS (Nishimoto et al., 2013). Significant recruitment of TDP-43 to paraspeckles might interfere with TDP-43-mediated RNA processing and disrupt RNA homeostasis in ALS motor neurons.

TDP-43 has been reported to co-localize with SG proteins, such as poly-A binding protein-1 (PABP-1; McGurk et al., 2014). Furthermore, several reports have demonstrated that TDP-43 localizes within SGs after stress treatment, including oxidative stress, heat shock and osmotic stress (Colombrita et al., 2009; Freibaum et al., 2010; Liu-Yesucevitz et al., 2010; Dewey et al., 2011). The recruitment of TDP-43 into SGs may be an initiating event, which could trigger pathological inclusion formation (Dewey et al., 2012; Li et al., 2013). In addition, TDP-43 inclusions are known to recruit important proteins into the inclusions and consequently affect their normal functions. For example, the RNA-binding motif 45 (RBM45) co-localizes with cytosolic TDP-43 inclusions in patients with ALS (Collins et al., 2012). Previous studies have suggested that RBM45 normally exists in nuclei, binds poly(C) RNAs and mediates the antioxidant response (Bakkar et al., 2015). Therefore, TDP-43 may regulate RNA homeostasis by controlling the amount of other RNA-binding proteins.

Mislocalization of TDP-43 has been reported in motor neurons of ALS patients (Arai et al., 2006; Neumann et al., 2006; Liu et al., 2015a). Giordana et al. (2010) have proposed the intriguing hypothesis that TDP-43 redistribution might be an early event in sporadic ALS). Indeed, mislocalization of TDP-43 in the cytoplasm during stress (e.g., oxidative stress, inflammation, energy dysfunction), as reported by several laboratories (Ayala et al., 2011; Correia et al., 2015; Liu et al., 2015a,b; Xia et al., 2016), might subsequently promote the formation of detrimental TDP-43 oligomers, decrease the normal amount of nuclear TDP-43, and cause detrimental effects. Of note, whether mislocalization of TDP-43 was observed in motor neurons of ALS-mSOD1 mouse models remains controversial. While no TDP-43 mislocalization was detected in motor neurons of three mSOD1 ALS mouse models (i.e., G93A, G37R and G85R; Robertson et al., 2007). Shan et al. (2009) observed that mislocalization of TDP-43 occurs in motor neurons of mSOD1-G93A mice having advanced disease). Importantly, redistribution of TDP-43 to the cytoplasmic region in motor neurons was observed in familial ALS patients carrying mutations in SOD1 (Robertson et al., 2007). These seemingly contradictory findings suggest that mSOD1-ALS mouse models may not fully recapitulate human ALS.

Lim et al. (2012) first reported the importance of AMPK in ALS, by using several genetic experimental models, and suggested that suppression of AMPK activity is protective in ALS. Liu et al. (2015a) have further reported higher activity of AMPK in motor neurons of ALS patients and animals. Pharmacological and genetic approaches have demonstrated that AMPK activation positively contributes to TDP-43 mislocalization. Given that AMPK is a major energy sensor and is usually activated in cells when the cellular ATP level is low or the cells are stressed (Long and Zierath, 2006; Hardie, 2008), these studies link energy dysfunction with TDP-43 mislocalization, which inevitably impairs RNA homeostasis, owing to the altered amount or/and cellular localization of TDP-43 in motor neurons as described above. Given that mitochondrial defects have been implicated in ALS and AMPK activation in studies during the past two decades (Dupuis et al., 2004a; Duffy et al., 2011; Watanabe et al., 2016), further investigations into the role of AMPK and its downstream pathways involved in the mislocalization of TDP-43 and RNA homeostasis would pave the way for a better understanding of the initiation of sporadic ALS.

Collectively, depletion of nuclear TDP-43, either by mislocalization to the cytoplasmic region or/and chelation by paraspeckles/Neat 1, TDP-43 inclusion or SGs would induce the dysregulation of TDP43-related RNA targets and contribute to ALS pathology.

### FUS/TLS

In addition to TDP-43, FUS/TLS is another important disease-causing gene in ALS. FUS/TLS-containing inclusions are also found in ALS motor neurons (Bäumer et al., 2010; Huang et al., 2010; Yamashita et al., 2012). FUS/TLS consists of 526 amino acids and is mainly located in nuclei. Several mutant forms of FUS/TLS, which are associated with ALS, are located in the cytoplasmic region (Kwiatkowski et al., 2009; Vance et al., 2009). Similarly to TDP-43, FUS/TLS is an RNA-binding protein that mediates multiple cellular pathways, including transcriptional regulation, mRNA splicing and miRNA production. For example, FUS/TLS interacts with several nuclear hormone receptors and modulates their functions (Powers et al., 1998). FUS/TLS also acts as a co-modulator for certain transcription factors (including NF-κB, SPI1 and RUNX (Runbox) transcription factor; Hallier et al., 1998; Uranishi et al., 2001; Li et al., 2010). Genome-wide approaches have revealed more than 5000 human RNA targets and more than 8000 mouse RNA targets for FUS/TLS (Lagier-Tourenne et al., 2012; Rogelj et al., 2012). Because FUS/TLS is a component of the hnRNP complex, it plays an important role in the splicing mechanism (Iko et al., 2004) and may alter the splicing patterns of more than 900 mRNAs (Lagier-Tourenne et al., 2012). Biochemical evidence has suggested that FUS/TLS regulates several mRNA targets that are important for spine and dendritic morphology (Fujii et al., 2005). Down-regulation of FUS/TLS causes abnormal synaptic function. Moreover, FUS/TLS is a component of the microprocessor complex and mediates the biogenesis of miRNA (Gregory et al., 2004). Similarly to loss of TDP-43, loss of FUS/TLS nuclear function is accompanied by the cytosolic inclusion of FUS/TLS in ALS motor neurons (Kwiatkowski et al., 2009; Vance et al., 2009).

During stress, FUS/TLS is recruited into SGs and co-localizes with an SG marker, PABP-1 (Andersson et al., 2008; Gal et al., 2011). Importantly, FUS/TLS with an ALS-linked mutation in its nuclear localization signal (NLS) causes the FUS/TLS variant to localize to the SGs more often (Bosco et al., 2010; Dormann et al., 2010; Ito et al., 2011). Like TDP-43, FUS/TLS also binds to several mRNAs of ALS-related genes (including VCP, VAPB, ubiquilin-2 and OPTN, Hoell et al., 2011; Colombrita et al., 2012; Lagier-Tourenne et al., 2012) and further modulates their expression. In addition, FUS/TLS is a key factor that regulates NEAT 1 stability and maintains nuclear body structure (Shelkovnikova et al., 2014). Interaction between FUS/TLS and NEAT 1 may contribute to the development of neuronal dysfunction in ALS.

Interestingly, activation of AMPK with AICAR (an AMPK activator, Corton et al., 1995) has been found to induce mislocalization of FUS/TLS in a motor neuron cell line (Lim et al., 2012; Liu et al., 2015a, b). Thus, similarly to TDP-43 (Liu et al., 2015a), abnormal activation of AMPK caused by impaired energy homeostasis in motor neurons might substantially alter RNA homeostasis by changing the cellular distribution of FUS/TLS.

### C9ORF72

The hexanucleotide repeat expansion of C9ORF72 is a frequent mutation observed in patients with ALS (5%–20% of sporadic ALS and 20%–50% of familial ALS; Boeve et al., 2012; Chio et al., 2012; Cooper-Knock et al., 2012). Patients with the C9ORF72 repeat expansion usually have earlier disease onset and accelerated disease progression, as compared with patients without the C9 repeat expansion (Byrne et al., 2012; Chio et al., 2012; Millecamps et al., 2012). The (G4C2) expansion transcripts are produced from the non-coding exons 1a and 1b of the C9ORF72 gene (C9, Gunnarsson et al., 1991) and the repeat number can be expanded to 700–1600 copies (DeJesus-Hernandez et al., 2011; Gijselinck et al., 2012). Although the function of the C9ORF72 gene remains unclear, expansion of the G4C2 repeats induces degeneration of motor neurons. Bioinformatic analysis has suggested that the C9ORF72 protein may be involved in autophagy and membrane trafficking (Zhang et al., 2012; Levine et al., 2013). Ciura et al. (2013) have shown that a decrease in C9ORF72 in zebrafish causes a locomotion deficit and reduces axon length). In addition, the G4C2 transcript frequently co-localizes with TDP-43 inclusions (McGurk et al., 2014).

RNA foci of the C9ORF72 repeat expansion were initially observed in the spinal cord of ALS/FTD patients (DeJesus-Hernandez et al., 2011; Renton et al., 2011), although the RNA level of C9ORF72 is decreased in patients with ALS and the C9ORF72 repeat expansion (Renton et al., 2011). The formation of RNA foci facilitates the recruitment of RNA-binding proteins and thus might interfere with their normal functions (Miller et al., 2000; Simón-Sánchez et al., 2012). For example, G4C2 RNA repeats interact with hnRNP-H, which mediates TARBP2 RNA splicing. Exogenous expression of G4C2 repeats decreases the levels of TARBP2 transcripts. This finding indicates that the C9ORF72 repeat expansion induces RNA toxicity by disrupting the function of hnRNP-H (Lee et al., 2013). Formation of the C9ORF72 repeat expansion-containing RNA foci also sequesters hnRNP-A3 and represses its RNA processing function (Mori et al., 2013a). Down-regulation of C9ORF72 RNA by antisense oligonucleotides decreases the number of RNA foci and RNA toxicity in human motor neurons derived from induced pluripotent stem cells (iPSCs) of ALS patients (Donnelly et al., 2013; Lagier-Tourenne et al., 2013). Collectively, these studies suggest that the C9ORF72 repeat expansion disturbs cellular RNA homeostasis by formation of RNA foci that recruit RNA-binding proteins. In addition, similarly to TDP-43, the C9ORF72 repeat expansion has been found in PABP-1-containing SG in patients with ALS (McGurk et al., 2014).

**Figure 1:**
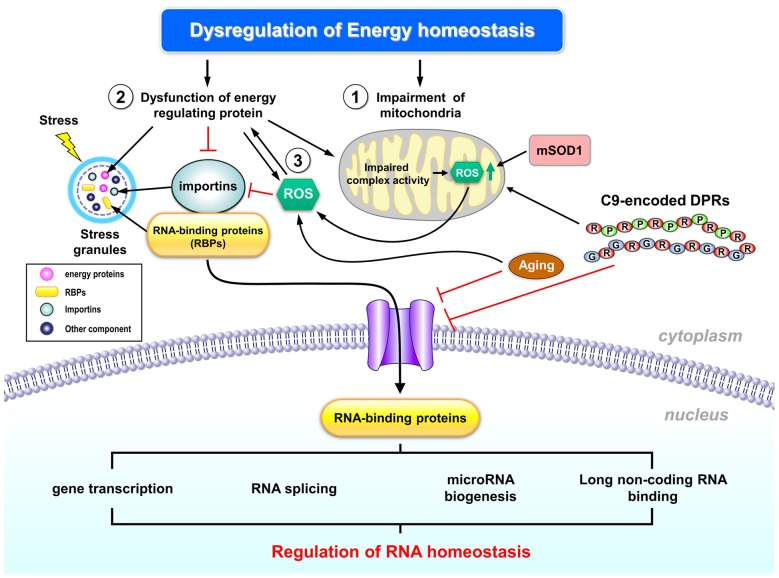
**Schematic representation of the potential regulation of RNA homeostasis by abnormal energy metabolism in amyotrophic lateral sclerosis (ALS).** Dysregulated energy homeostasis might result from dysregulation of mitochondrial function, which has been well documented in ALS pathogenesis (1). Many ALS- associated genes (including TDP-43, FUS/TLS and C9ORF72) have been implicated in the regulation of mitochondrial functions and RNA metabolism, thus suggesting that altered mitochondrial functions and RNA toxicity might contribute to ALS pathogenesis. In addition, dysfunction of energy-regulating proteins may also contribute to dysregulated energy homeostasis in ALS (2). Specifically, recent studies suggest that abnormal cellular energy status in ALS enhances activity of an energy sensor (AMP kinase, AMPK) that interferes with the transport of several RNA-binding proteins (RBPs) into nuclei of motor neurons, which might be one of the early events in ALS pathogenesis. The impaired transport of nuclear RBPs might disrupt cellular RNA homeostasis. Moreover, reactive oxygen species (ROS) production might also cause the abnormal activation of energy regulating proteins (3), and subsequently led to the mislocalization of RBPs. Further investigation is required to delineate the potential regulation of RNA homeostasis by energy dysfunction, to explore the key players involved in such regulation, and to consolidate the role of such abnormality in ALS pathogenesis. See text for additional details.

Importantly, C9ORF72 repeat expansion had been implicated in the impairment of nuclear import by interacting with a Ran GTPase-activating protein (RanGAP) in a *Drosophila* model and in motor neurons derived from iPSCs of C9-ALS patients. This impairment of nuclear transport can be rescued by antisense oligonucleotides against the (G4C2) G-quadruplexes, thus suggesting the importance of the hexanucleotide repeat expansion (Zhang et al., 2015). Another study also suggested that G4C2 repeat expansion causes the abnormalities of the nuclear envelope and results in dysfunction of nucleocytoplasmic transport (Freibaum et al., 2015). Consistent with the importance of nucleocytoplasmic transport in ALS, screening for modifiers of C9ORF72 toxicity using either *Drosophila* or yeast also identified a group of genes (such as importins, proteins of nuclear pore complex and Ran-GTP cycle regulators) involved in nucleocytoplasmic transport (Jovičić et al., 2015; Boeynaems et al., 2016a; Figure 1). Moreover, the C9ORF72 repeat expansion also induces protein toxicity by the repeat-associated non-ATG translation (RAN translation), which allows C9ORF72 repeat transcripts to be translated into dipeptide repeat (DPR) proteins consisting of di-amino acids. These DPRs contain poly-(glycine-alanine, GA), poly-(glycine-proline, GP), poly-(glycine-arginine, GR) and poly-(proline-alanine, PA) and form pathological inclusions in ALS (Mori et al., 2013b). Notably, impairment of nuclear transport by one specific DPR aggregate (i.e., poly-GA) has recently been shown to cause TDP-43 mislocalization in primary neurons (Khosravi et al., 2017), which further worsen RNA toxicity as described above. These findings further highlight the important role of nucleo-cytoplasmic transport in C9-ALS.

Expression of the C9ORF72 repeat expansion and the resultant DPRs is associated with abnormal mitophagy and impaired mitochondrial function (Lopez-Gonzalez et al., 2016; Onesto et al., 2016), thus supporting the potential involvement of energy dysregulation in C9-associated ALS. Further investigations are required to evaluate whether and how the RNA toxicity and energy dysfunction caused by C9ORF72 repeat expansion cross-regulate each other.

### Importins and AMPK in Nucleocytoplasmic Transport

As noted by several recent studies and reviews, the loss of nuclear functions of many RNA-binding proteins and nuclear transport components and the gain of function and/or aggregate formation of these proteins in the cytoplasm may contribute to ALS pathogenesis (Zhang et al., 2015, 2016; Boeynaems et al., 2016a,b; Jovičić et al., 2016; Taylor et al., 2016). Mutations in several major ALS genes (e.g., TDP-43, FUS/TLS) and the aging process appear to trigger these progressive impairments (Jovičić et al., 2016). Recent studies have demonstrated that aggregates located in the cytoplasm are likely to interfere with nucleocytoplasmic transport functions (Khosravi et al., 2017; Woerner et al., 2016). Nucleocytoplasmic transport has become one of the most heavily researched topics in ALS pathogenesis, because the disturbance of this process might be the crucial and initial step that triggers ALS pathogenesis in motor neurons (Dormann and Haass, 2011). Among all of the proteins involved in nucleocytoplasmic transport, we are most interested in importins because they are regulated by AMPK (Wang et al., 2004).

Importins are key molecules in the control of nucleocytoplasmic transport because they are needed for transporting macromolecules larger than 40 kDa (such as proteins; Görlich and Kutay, 1999; Depping et al., 2015). Most proteins transported by importin α/β contain specific NLSs. A total of six different NLS classes have been reported, and each of them binds to a different binding site on the importin α isoform (Kosugi et al., 2009). Interestingly, there are six importin α subtypes in mice, and seven importin α subtypes in human, but only one importin β is present in both organisms (Miyamoto et al., 2016). These importin α subtypes not only function in the control of nucleocytoplasmic transport but also have been implicated in mRNA biogenesis (Wen and Shatkin, 2000), and protein degradation (Kim et al., 2014).

Multiple lines of evidence suggest the involvement of importins in ALS. First, we have previously demonstrated that AMPK activity is higher in motor neurons of the ALS spinal cord when compared with that of non-ALS subjects (Liu et al., 2015a). Second, cellular localization of importin-α1 in the motor neurons of patients with ALS is altered. Most importantly, phosphorylation of importin-α1 by AMPK caused the mislocalization of importin and human antigen R (HuR, a major RNA stability factor, Nabors et al., 2001; Doller et al., 2008) in the cytoplasm (Liu et al., 2015b). Notably, HuR has been implicated in ALS (Lu L. et al., 2009; Lu et al., 2014). It is also intriguing that importins are located in SGs and have been implicated in ALS pathology by bioinformatic analysis (Boeynaems et al., 2016b). Mislocalization of HuR in motor neurons is closely associated with a lower level of VEGF in patients with ALS and is known to regulate the expression of TDP-43 and FUS/TLS (Lu et al., 2014). This is also an interesting finding because VEGF is known to protect against neurotoxicity and delay disease progression in a mouse model of ALS (Lladó et al., 2013; Wang et al., 2016). It is very likely that the AMPK-mediated phosphorylation of importin-α1 may lead to mislocalization of HuR, thus negatively affecting the normal function of HuR in RNA processing and eventually causing ALS pathology. For example, it has been demonstrated that mislocalization of HuR by abnormal activation of AMPK in a motor neuron cell line (NSC34) reduces the half-life of its target mRNA (i.e., VEGF; Liu et al., 2015b). This finding is consistent with an earlier study that mislocalization of HuR in motor neurons of ALS patients is closely associated with a lower level of VEGF (Lu et al., 2014). Together, these observations suggest that energy dysfunction in ALS might alter the cellular distribution of RBPs (such as HuR) and subsequently alter homeostasis of their target mRNAs (e.g., VEGF).

This alteration in the function of importin-α1 induced by AMPK phosphorylation is likely to be a common pathogenic pathway that may contribute, at least partially, to the RNA toxicity observed in ALS. In accordance with the importance of importins in ALS, up-regulation of importin α has been found to be beneficial in a fly model of ALS that exogenously expresses the C9ORF72 repeat expansion (Zhang et al., 2015). In contrast, suppression of importin β1 enhances the toxicity of DRPs (Boeynaems et al., 2016a). These data collectively suggest that importins and abnormal activation of AMPK are important therapeutic targets for ALS.

### RNA Transport

Proper RNA transport is critical for cellular RNA homeostasis. In highly polarized cells, such as motor neurons, mRNA can be transported to neurites. Motor neurons are known to have local protein synthesis in their synapses after exposure to stimuli (Yoo et al., 2010). Although TDP-43 and FUS/TLS are primarily considered to be nuclear proteins, several studies have suggested that these proteins have cytoplasmic functions, including local translation with synaptic plasticity, mRNA transport and RNA stabilization. Several studies demonstrated that cytosolic TDP-43 and FUS/TLS can transport mRNA to dendritic spines after stimulus exposure (Kanai et al., 2004; Belly et al., 2005; Fujii and Takumi, 2005; Wang et al., 2008). In addition, FUS/TLS may recruit mRNA to RNA granules and transport them to dendritic spines after activation of the metabotropic glutamate receptor 5 (mGluR5; Fujii et al., 2005). After post-synaptic activation of mGluR5, the FUS/TLS-containing complex may facilitate the activity of the actin-based motor by interacting with myosin (Yoshimura et al., 2006; Takarada et al., 2009). Similarly, cytoplasmic TDP-43 has been demonstrated to trigger dendrite formation (Feiguin et al., 2009; Lu Y. et al., 2009). The TDP-43-containing RNA complex is also known to interact with PSD-95 and to regulate neuronal activity (Wang et al., 2008). TDP-43 and FUS/TLS also regulate transport ribonucleoprotein particles (tRNPs; Sephton and Yu, 2015). Additional functions of TDP-43 include the stabilization of low molecular weight neurofilament mRNA by binding to the UG motif (Strong et al., 2007; Volkening et al., 2009) and the regulation of the transport and local translation of important transcripts (e.g., fragile mental retardation protein and Staufen) in neurons (Wang et al., 2008). Given that RNA transport to dendrites requires energy (Davis et al., 1987), dysregulation of energy homeostasis in motor neurons is expected to contribute to the RNA toxicity observed in ALS because the correct localization of RNAs is critical for neuronal activity. RNAs localize to dendrites and translation of these mRNAs are important for synaptic remodeling and plasticity (Martin and Zukin, 2006). The abnormal localization of RNAs caused by impaired energy homeostasis may disrupt RNA homeostasis and further cause RNA toxicity.

## Concluding Remarks

As described above, many ALS genes encode RNA-binding proteins (RBPs, Figure 1) that control RNA homeostasis and have been implicated in the regulation of mitochondrial function. During various cellular stresses (including elevated oxidative stress, impaired mitochondrial function, ER stress and aging), mislocalization of some RBPs (e.g., TDP-43, FUS/TLS and HuR) might occur in motor neurons. This aberrant cellular distribution of RBPs is likely to disrupt RNA homeostasis in motor neurons, and was proposed to be one of the early events in the development of ALS. Future investigations should further delineate the pathway(s) and explore the crucial molecular players underlying the potential regulation of RNA homeostasis by energy dysfunction, and consolidate whether such abnormality plays an important role in ALS pathogenesis.

## Author Contributions

Y-JL wrote the first draft from section Abnormal Energy Homeostasis in ALS to section *C9ORF72*, P-YT wrote the first draft of section *Importins and AMPK in Nucleocytoplasmic Transport* and RNA Transport, YC organized the manuscript and wrote the final draft.

## Conflict of Interest Statement

The authors declare that the research was conducted in the absence of any commercial or financial relationships that could be construed as a potential conflict of interest.
